# Rapid Color Quality Evaluation of Needle-Shaped Green Tea Using Computer Vision System and Machine Learning Models

**DOI:** 10.3390/foods13162516

**Published:** 2024-08-12

**Authors:** Jinsong Li, Qijun Li, Wei Luo, Liang Zeng, Liyong Luo

**Affiliations:** 1 Integrative Science Center of Germplasm Creation in Western China (CHONGQING) Science City, College of Food Science, Southwest University, Chongqing 400715, Chinally1979@swu.edu.cn (L.L.); 2 Chongqing Key Laboratory of Speciality Food Co-Built by Sichuan and Chongqing, Southwest University, No. 2 Tiansheng Road, Beibei District, Chongqing 400715, China; 3 College of Food Science, Southwest University, No. 2 Tiansheng Road, Beibei District, Chongqing 400715, China; 4 College of Computer and Information Science, Southwest University, No. 2 Tiansheng Road, Beibei District, Chongqing 400715, China

**Keywords:** green tea, color characteristic, computer vision system, machine learning

## Abstract

Color characteristics are a crucial indicator of green tea quality, particularly in needle-shaped green tea, and are predominantly evaluated through subjective sensory analysis. Thus, the necessity arises for an objective, precise, and efficient assessment methodology. In this study, 885 images from 157 samples, obtained through computer vision technology, were used to predict sensory evaluation results based on the color features of the images. Three machine learning methods, Random Forest (RF), Support Vector Machine (SVM) and Decision Tree-based AdaBoost (DT-AdaBoost), were carried out to construct the color quality evaluation model. Notably, the DT-Adaboost model shows significant potential for application in evaluating tea quality, with a correct discrimination rate (CDR) of 98.50% and a relative percent deviation (RPD) of 14.827 in the 266 samples used to verify the accuracy of the model. This result indicates that the integration of computer vision with machine learning models presents an effective approach for assessing the color quality of needle-shaped green tea.

## 1. Introduction

Green tea is widely consumed around the world due to its pleasant taste and potential health benefits [1,2]. Chongqing, a city located in southwestern China, is recognized as a potential origin of tea [3]. Chongqing lies in the prime region for green tea cultivation and is celebrated for its bountiful yield of needle-shaped green tea. Notably, Yongchuan Xiuya is recognized as a unique Chinese agricultural specialty valued nationwide for its straight appearance, light green color, delicate and aromatic profile, as well as its umami taste. Additionally, it represents one of China’s most quintessential needle-shaped green teas. Therefore, developing a swift, objective, and precise assessment approach for evaluating the quality of needle-shaped green tea is significant for the tea industry in Chongqing and nationwide. When evaluating the quality of tea, several attributes are considered, including appearance, aroma, taste, and other sensory factors [4]. Among these attributes, the appearance, particularly the color, plays a crucial role in green tea as it greatly influences purchasing decisions [5]. Traditional methods of assessing tea quality rely on sensory evaluation and quantitative descriptive analysis [6]. However, these techniques require specialized training and have a steep learning curve, resulting in high costs, time-consuming processes and inaccuracy [7]. Consequently, consumers often associate higher prices with higher quality due to a lack of standardized evaluation methods [8]. Therefore, it is essential to establish an accurate, efficient, and objective approach to tea quality evaluation, specifically in terms of assessing appearance. Implementing such standardization would provide valuable guidance to consumers in the market.

### 1.1. Related Works and Motivation

In recent years, there has been a growing utilization of various sensors, including computer vision [9,10], near-infrared spectroscopy [11], and electronic tongues and noses [12], etc., in the realm of food detection. This trend has fueled the rapid advancement of technologies for fast, accurate, non-destructive detection and assessment, which are presently being studied and applied in the tea industry. These technologies find broad application in monitoring fresh leaves [13], processing processes [14], evaluating tea quality [15]. However, the assessment of the color of tea continues to rely on relatively traditional methods; sensory evaluation [16] and colorimeter assessment methods [17] continue to serve as the primary means of evaluation. Hence, it is essential to develop a fast, objective, and precise assessment method for the color of tea. Computer vision is a field that focuses on the theoretical and algorithmic foundations for deriving meaningful information about objects from images [18]. With its inherent qualities of accuracy and objectivity, it has proven to be a valuable tool for assessing the quality of various products [19], including tea. Ren [20] showcased the application of computer vision technology in the extraction of the morphological parameters of Congou black tea, resulting in the efficient identification of different tea quality grades. In a similar vein, Xu [21] integrated E-nose technology with computer vision to enable rapid and precise determination of the quality grades of Longjing tea. This innovative approach presents a novel idea for achieving high-precision tea quality assessment. High accuracy requires high-performance algorithms, with machine learning playing a pivotal role. In recent studies focused on tea-related topics, numerous machine learning models have demonstrated remarkable success. Widely used machine learning models, such as Support Vector Machine and Random Forest, have exhibited notable advantages in various tasks, including the management of tea plantations [22], processing monitoring [23], tea quality assessment [24], and rapid material detection [25]. Decision Tree (DT) is a simple but powerful classifier that can handle both discrete and continuous features. DT-based Adaboost (DT-Adaboost) can give full play to the classification ability of DT and improve the overall classifier performance [26]. Therefore, DT-Adaboost is well adapted to handle complex data. DT can handle complex situations such as nonlinear relationships, interactions and high-dimensional features, so DT-Adaboost can achieve good classification results when dealing with complex data sets. Meanwhile, DT-Adaboost can reduce the risk of overfitting by iteratively training multiple DT weak classifiers and combining them in a weighted way [27]. For tea images, it is necessary to use DT-Adaboost to improve the performance of tea image recognition in terms of feature complexity and feature similarity. Furthermore, it is important to note that these studies relied on price-based grading of the samples instead of assessments based on sensory evaluations, which may pose challenges in accurately representing the true quality of tea. Additionally, these studies did not evaluate the color characteristics of dried green tea, which is an important aspect of tea quality assessment.

### 1.2. Contributions and Paper Organization

The objective of this research is to develop a precise, efficient, and unbiased technique for assessing the quality of needle-shaped green tea based on its color characteristics. To achieve this goal, a comprehensive dataset consisting of 885 images was compiled, representing 157 distinct samples of green tea from Chongqing. In order to prepare the images for analysis, preprocessing techniques such as clipping and grayscale transformation were applied. Subsequently, RGB, HSV, and Lab color features were extracted from the processed images. Using sensory evaluation outcomes as ground truth labels, the research focused on constructing an optimal identification model utilizing support vector machine (SVM), Random Forest (RF), Decision Tree with Adaptive Boosting (DT-Adaboost) integrated learning models. By providing both theoretical foundations and empirical evidence, this study aims to contribute to the digitization and standardization of tea quality assessment practices, regulating the tea market, and enhancing consumer experiences.

The rest of this paper is organized as follows. In Section 2, we outline the materials and methodologies utilized in this study, encompassing tea samples, sensory evaluation techniques, image acquisition and processing approaches, as well as the model training and validation procedures. In Section 3, we conduct a comparison of algorithm performance across different tasks and subsequently discuss the outcomes, and finally, we conclude the paper in Section 4.

## 2. Materials and Methods

### 2.1. Sample Preparation

Green tea samples were purchased from various companies located in Chongqing, China. All the samples were processed using the needle-shaped green tea processing method. The standard processing procedures included spreading, fixing, rolling, initial drying, carding, and final drying. Given that color is influenced by fluctuations in moisture content [28], all the green tea samples utilized in this study conformed to the Chinese National Official Standard GB/T 14456.1-2017 [29] (Green tea-Part 1: Basic requirements) for needle-shaped green tea, with a moisture content of less than 7%; the precise moisture content data for each sample can be found in Table S1. A total of 157 samples were obtained over a two-month period (March and April 2022). In order to preserve the color quality of the green tea samples, each sample was sealed in an aluminum foil bag and stored at 4 °C until the analysis process.

### 2.2. Sensory Evaluation Methods

**Figure 1:**
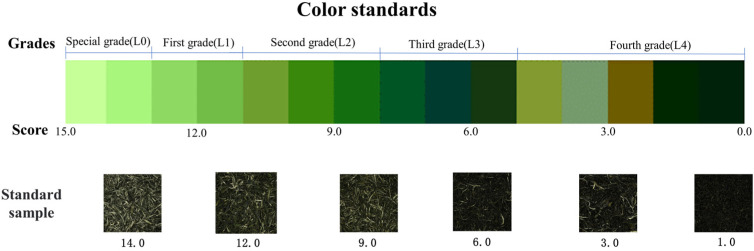
Color standards used for the color sensory evaluation of green tea.

In the initial stage, color cards (Figure 1) for sensory evaluation were prepared as described, which involved gathering images of all the standard samples and extracting lab parameters from these images. These parameters were then used to determine the starting and ending values of the color scale. These ranges were separated by 10 intervals, respectively. From these defined ranges, the color blocks that most accurately depicted the transition from light green to dark brown were chosen to construct the color card. Thus, objective lab parameters were linked to sensory quality [30]. A team of eight evaluation experts collaborated to perform this step, and the specific lab parameters are displayed in Table S2.

The color qualities of green tea were assessed according to the guidelines specified in the Chinese National Official Standard GB/T 23776-2018 [31], with particular emphasis on the color of dried tea leaves. A reference color card was utilized to aid in color evaluation (Figure 1). The color characteristics of 157 varieties of green tea were assessed by a skilled panel consisting of 8 highly trained panelists from the College of Food Science at Southwest University (Chongqing, China). Each panelist underwent 20 training sessions lasting 1.5 h before each experiment. Before taking part, all participants provided written informed consent [32]. The performance assessment of panelists in the color evaluation group is depicted in Figure S1. Approximately 50 g of each sample was placed in the evaluation tray [24], and a panel of 8 professionally trained panelists assessed the color characteristics. The average score from the panelists was then used as the sample’s color score, and subsequent grading was assigned based on these scores. This study was approved by the Ethics Committee of Southwest University (IRB Number: H23151).

### 2.3. Image Acquisition

The computer vision system was configured with a specialized industrial camera, (ME-EM500C, Shanxi Microvision Intelligent Manufacturing Technology Co., Ltd., Xi’an, China). This camera is designed for high-performance image acquisition in industrial settings. Additionally, a uniform light source (MV-ALP2410, Shanxi Microvision Intelligent Manufacturing Technology Co., Ltd., Xi’an China) was incorporated to ensure consistent lighting during image capture. The system was housed in a darkroom to minimize external interference and ensure accurate image processing. A graphical user interface (GUI) software(Microvision x64 4.0.18.610B, from Shanxi Microvision Intelligent Manufacturing Technology Co., Ltd., Xi’an, China) processing system was utilized to facilitate easy control and analysis of the captured images. The GUI software interface and its associated functions are shown in Figure S2. Together, these components formed a robust and reliable computer vision system for sample image acquisition. 

**Figure 2:**
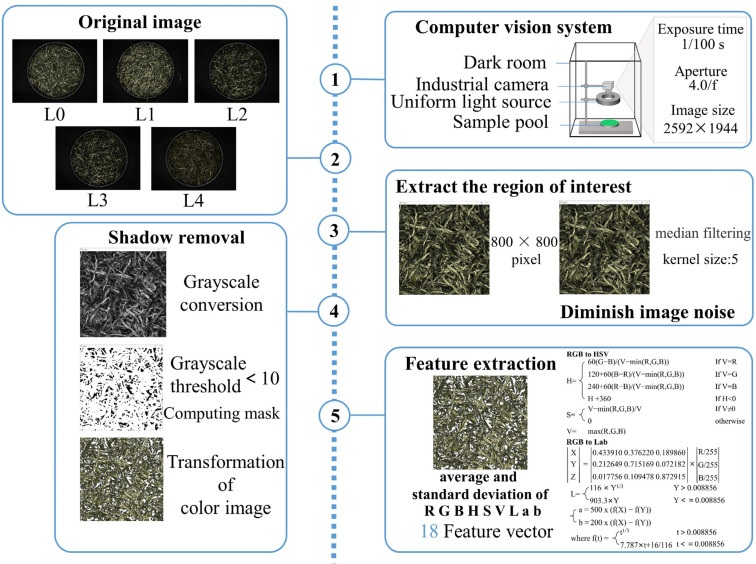
Flow chart of the image acquisition and feature extraction of tea samples.

To conduct the image acquisition, 20 ± 0.5 g of tea was extracted from each sample and evenly distributed in the sample pool (Petri dishes) [33]. The sample pool was then placed under the uniform light source to capture images. It should be noted that all images were taken using the camera settings detailed in Figure 2. The specific camera characteristics and lighting conditions are provided in Table S3. To minimize the impact of internal variations among the samples on the results, each sample was randomly sampled five times, resulting in a total of 785 images.

During our sample collection, we observed an imbalance in the number of green tea samples categorized as special grade and fourth grade compared to other categories. This is in line with the proportion of tea product quality distribution in the market, but this data imbalance poses a challenge to the effectiveness of model training. To address this issue, we performed data augmentation by supplementing the green tea images of special grade and fourth grade. Data imbalance is a common occurrence [34], and data augmentation can mitigate this issue. Geometric transformation can be used as a data augmentation technique, is known for its ease of implementation and has been shown to enhance model performance effectively in recent studies in the food-related domain [35,36]. Therefore, we applied various transformations to each green tea image, including rotations of 90, 180 and 270, as well as horizontal flipping [34]. This data enhancement strategy aims to rectify the imbalance in the dataset, ensuring that the model is exposed to a more representative distribution of green tea samples across different grades. By applying these transformations, we generated four additional images for each original image. As a result, the dataset was expanded from its original size of 785 images to a total of 885 images after data augmentation. 

### 2.4. Feature Extraction

As shown in Figure 2, the image underwent preprocessing to extract an 800 × 800-pixel region of interest (ROI) centered on the raw image’s central pixel. Median filtering was applied to diminish image noise [37]. Subsequently, the image required shadow removal. The shadow is caused by underexposure of the lower leaves due to light projecting onto the surface of the above table during the time of shooting. This shadow removal is accomplished by automatically applying a pre-defined grayscale threshold [38]. The OpenCV-Python library is utilized for extracting color features from the region of interest. The mean and standard deviation values were calculated for each channel within the RGB, HSV, and CIELab color spaces. As a result, a total of 18 color features were acquired, which include the average and standard deviation of the red channel (Ra, Rs), green channel (Ga, Gs), blue channel (Ba, Bs), hue channel (Ha, Hs), saturation channel (Sa, Ss), value channel (Va, Vs), a component channel (aa, as), b component channel (ba, bs), and lightness component channel (La, Ls). To assess the validity of the color variables, a Pearson correlation analysis was performed between these variables and the sensory score using the SPSS software (Statistics 25.0.0, IBM, Armonk, NY, USA).

### 2.5. Model Establishment and Performance Evaluation

**Figure 3:**
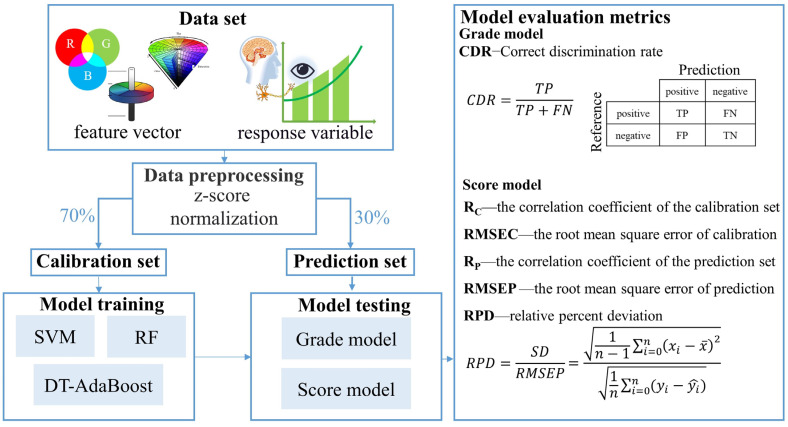
Flow chart of the model training and validation process.

Machine learning was used to build predictive models for sensory quality of the green tea. Three efficient machine learning models were used to predict the tea quality, including SVM, RF, and DT-AdaBoost (Figure 3).

SVM is a widely applied machine learning model in scientific research and industry [20]. The basic concept of the model is to seek a classification hyperplane that can divide the training dataset into a discrete predefined number of classes as the decision surface [20]. In theory and in application, the SVM can obtain accurate classification results by two key parameters, including kernel function and penalty coefficient (c). In this study, we use Radial Basis Function (RBF) as the kernel function of SVM; thus, the key parameters of the model are the optimization penalty coefficients (c) as well as the gamma of the RBF kernel function (g). In order to maximize the accuracy of the model, we used the grid search method to search the parameter pairs (c, g) in the range of 0.01–100.

RF [39] is an ensemble learning method which consists of multiple decision trees and uses bootstrap aggregating (bagging). Each decision tree is constructed independently and results in a final classification result or regression value based on majority voting or average prediction [40]. The RF can obtain accurate classification results by two key parameters, including maximum depth of decision tree (depth) and number of estimators (N). The number of estimators denotes the number of decision trees in a random forest. When optimizing the parameters using the grid search method, we set the range of the parameter pairs (depth, N) to 1–12 and 1–1000.

The SVM and RF models have been widely employed in research on evaluating tea quality, demonstrating favorable outcomes in tea classification. In more extensive application contexts and with increased accuracy demands, we endeavored to employ decision tree models for tea quality evaluation to examine their efficacy in both classification and regression undertakings. DT-AdaBoost is another ensemble learning method. It can be used in conjunction with many other types of weak learners to improve performance. Weak learners are a set of learning algorithms or models that individually perform relatively poorly in terms of accuracy or predictive power. In previous studies, the weak learner of AdaBoost is usually a decision tree [41]. Thus, we also chose Decision Tree-based AdaBoost (DT-AdaBoost) as the prediction model. Because the same weak learner as RF is chosen, the key parameters of DT-AdaBoost are the same as those for the RF, including the maximum depth of decision tree (depth) and number of estimators (N), and we also set the search range to 1–12 and 1–1000.

Before establishing the model, the color data set was divided into calibration and prediction sets in the ratio of 7:3 [42]. The objective is to prevent overfitting resulting from additional dataset partitions [43]. Prediction models for color sensory quality grades and scores were then established using SVM, RF, and DT-Adaboost algorithms. Prior to this, the Z-score method [14,44] was applied to preprocess the original data.

The accuracy of the grade model was assessed using the correct discrimination rate (CDR), defined as the ratio of correctly estimated samples to the total number of samples. A higher CDR value indicates a more effective discriminant model. For evaluating the score evaluation model, we employed several indices, including the Correlation Coefficient of the calibration set (Rc), Root Mean Square Error of Cross Validation (RMSECV), Correlation Coefficient of the prediction set (Rp), Root Mean Square Error of Prediction (RMSEP), and the Relative Percent Deviation (RPD). A scoring model with larger Rc, Rp, and RPD values, as well as smaller values of RMSECV and RMSEP, reflects superior prediction performance [44].

## 3. Results and Discussion

### 3.1. Sensory Evaluation and Color Feature Extraction Analysis

The sensory evaluation results revealed a wide range of scores, ranging from 3 to 13, indicating the diverse quality grades present in the 157 samples collected for this study. This variability suggests that the data are representative and can be relied upon for thorough analysis and drawing conclusions.

The evaluation of the quality of tea is heavily influenced by the color of dried tea leaves, as it plays a significant role in the initial impression formed by consumers when making purchasing decisions [5]. In order to move towards a more objective assessment method and reduce reliance on subjective sensory evaluation, it is important to explore the relationship between objective physicochemical indicators and sensory quality. The RGB color model, which captures the intensity of light in the red (R), green (G), and blue (B) spectrum, is widely used for color representation [45]. The HSV color space is characterized by H (hue), S (saturation), and V (value), closely mirroring human perception of colors [46]. Furthermore, for color measurements, the Lab* (CIELab) color space is internationally recognized as the standard and is extensively applied in assessing the color of various food products [46]. Therefore, our goal is to provide an unbiased assessment of the color quality of green tea based on these color characteristics.

**Figure 4:**
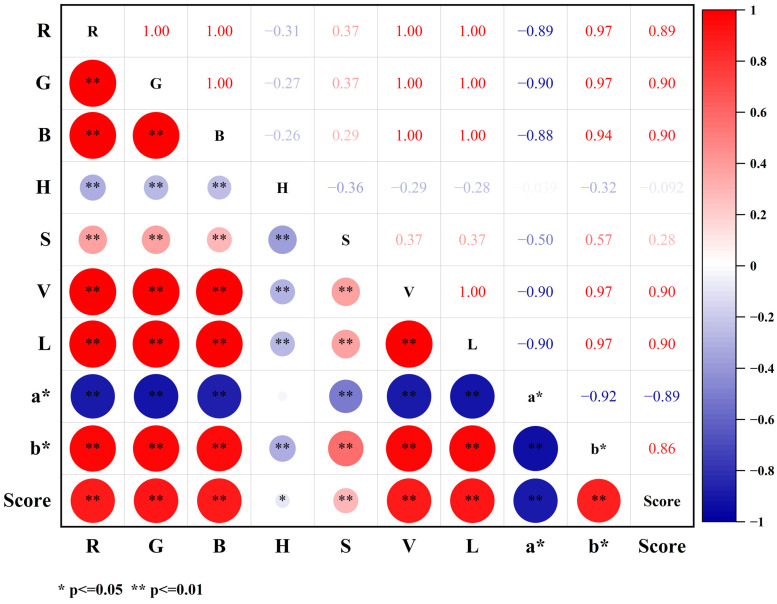
Heatmap for Pearson’s correlation coefficients between color characteristics and sensory quality.

Pearson’s correlation coefficients (r) and two-tailed tests were conducted to analyze the relationship between the color features of green tea and sensory quality, as depicted in Figure 4. The color characteristics of the green tea exhibited significant correlations with sensory qualities. Specifically, the R, G, B, S, V, L, and b* values showed negative correlations, while the a* and H values demonstrated positive correlations with sensory qualities. These findings suggest that greener color and higher saturation levels are generally associated with better sensory qualities, confirming the findings of previous research [47].

**Figure 5:**
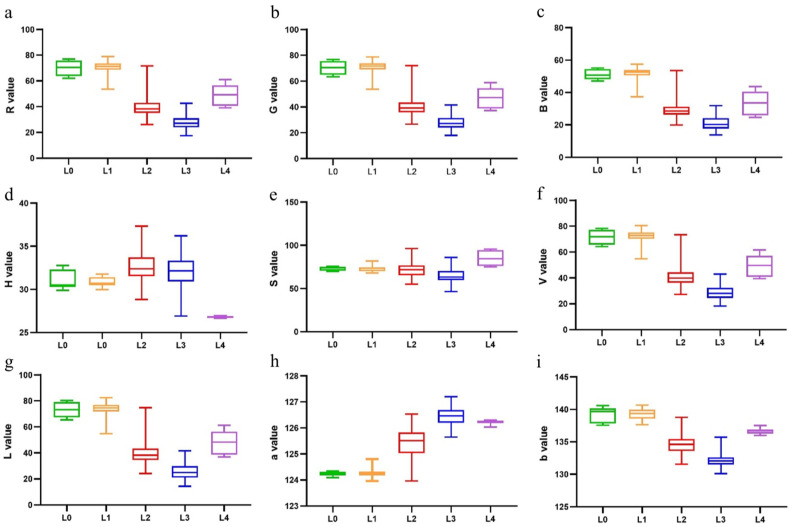
Box plots of image color characteristics: (**a**) R; (**b**) G; (**c**) B; (**d**) H; (**e**) S; (**f**) V; (**g**) L; (**h**) a; and (**i**) b.

To illustrate the overall distribution of data, the comparison of different grades of color variables, such as RGB, HSV, and Lab, is presented in Figure 5. The color features of the first four grades exhibit a general downtrend except for a*, which demonstrates a consistent upward trend. This trend resembles the color changes observed during processing [48]. Therefore, implementing improved techniques can help alleviate darkening and prevent the loss of luster in green tea processing, ultimately leading to higher-quality green tea products. Notably, the fourth-grade tea samples demonstrate a wide range of values in color characteristics other than H, a*, and b*. A low and concentrated H value suggests a color leaning more towards red, orange, or yellow, and less associated with green, cyan, or blue [46]. The concentrated ab values suggest similar hues and potential affiliation with a closely related color group. The substantial variation in brightness and contrast leads to the display of various unappealing color traits in the tea samples, such as gray–green, sere yellow, and dark brown, in line with the outcomes of sensory evaluation.

### 3.2. Grade Evaluation Model Results

In this study, the evaluation of grades was performed as a classification task in machine learning. SVM, RF, and DT-AdaBoost were used to build classifiers to establish evaluation models for grading tea quality based on color. The purpose was to demonstrate the effectiveness of machine learning classification models in color grade evaluation. These models were implemented using the scikit-learn library in Python.

**Figure 6:**
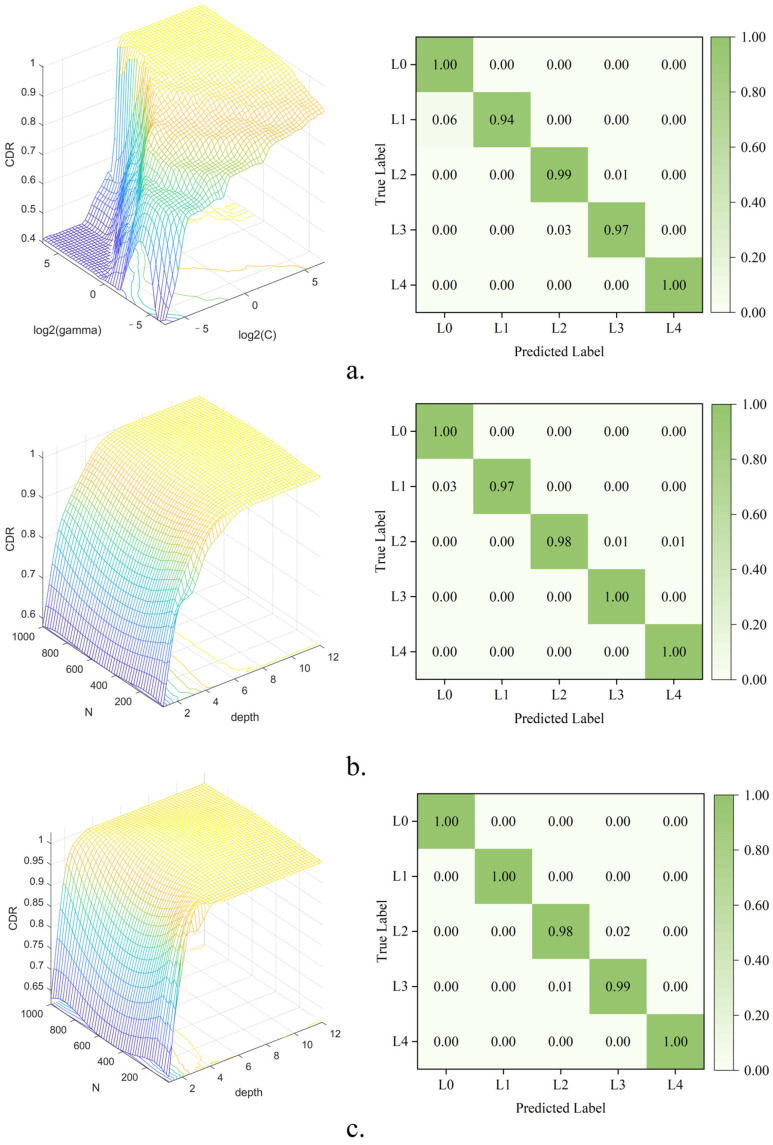
Optimization of parameters for grade prediction models and the confusion matrix of different machine learning methods: (**a**) SVM; (**b**) RF; and (**c**) DT-Adaboost.

Considering that all three models have key parameters that impact the model results, the grid search method was used to optimize these models to obtain the optimal parameters. The results of the hyperparameter search are shown in Figure 6. Table 1 presents the classification results for estimating the color of dried green tea using the various machine learning models. The best result after hyperparameter search was used for each model. As shown in Table 1, all the models demonstrated high accuracy in assessing the color grades of tea. Specifically, the SVM model achieved a CDR of 97.74%, while the RF and DT-Adaboost models achieved CDRs of 98.12% and 98.50%, respectively, in the prediction set. Notably, the DT-Adaboost model achieved the optimal discrimination results. The confusion matrixes of the models are shown in Figure 6. The confusion matrixes indicate that the recall value of L0 and L4 is nearly 100%, which means that there is no misjudgment. The misjudgments mainly occur between L1, L2 and L3. A comparison of the accuracy, precision, and F1 score data reveals that the performance of the three algorithmic models in grade evaluation is closely matched. The identified features are capable of effectively discerning varying grades of tea based on distinct color characteristics.

### 3.3. Score Evaluation Model

**Figure 7:**
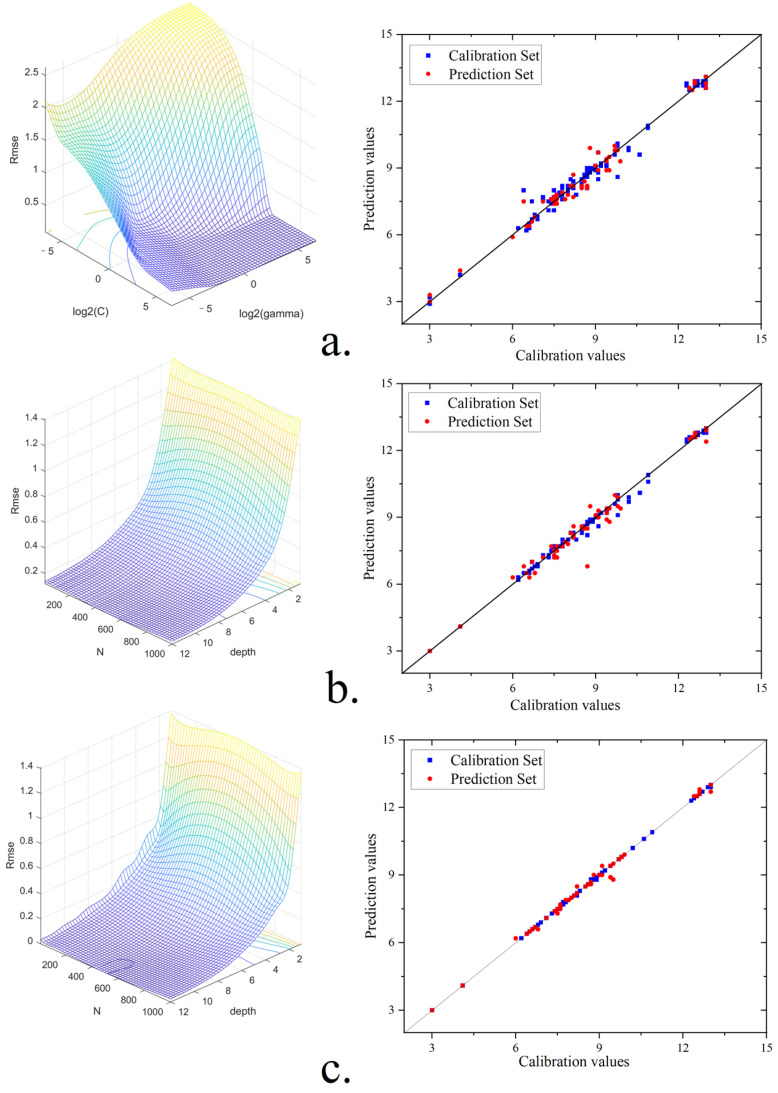
Optimization of parameters for score prediction models and the scatter plot of the predicted and actual values from the sensory model: (**a**) SVM; (**b**) RF; and (**c**) DT-Adaboost.

For score evaluation, our approach is to use a regression task in machine learning. To predict the color sensory scores of green tea, we constructed SVM, RF, and DT-AdaBoost as regressors to establish evaluation models. The methods of model establishment and hyperparameter search are consistent with those in Section 3.2. The results of the hyperparameter search are shown in Figure 7.

The parameters that correspond to the lowest RMSEC value were selected for model training after the hyperparameter search. As shown in Table 2, the SVM, RF, and DT-AdaBoost regression models had respective RMSEC values of 0.313, 0.296, and 0.162, and corresponding RPD values of 7.668, 8.122, and 14.827. These results indicate that all three models exhibit good predictive performance. Notably, the DT-AdaBoost model had the lowest RMSEP value and the highest RPD, with Rp, Rc, and RMSEC values of 0.995, 0.999, and 0.020, respectively.

DT is one of the most widely used machine learning algorithms for solving classification and regression problems [49]. Its versatility and effectiveness make it particularly suitable for the evaluation tasks involved in grading and scoring tea quality. Figure 7 includes the results of score prediction for the samples from different models in the score evaluation, along with the true score results. In Figure 7, the red dots represent the calibration set samples, and the blue dots represent the prediction set samples. The closer the distribution of the dots is to the diagonal line, the closer the predicted value is to the true value. Figure 7 shows that the distribution of red and blue dots of DT-AdaBoost is closest to the diagonal line, followed by RF and SVM. 

### 3.4. Discussion of the Models’ Results

To thoroughly assess the quality of tea, it is important to take into account multiple aspects, such as various features, classifications, and regressions. This highlights the necessity of utilizing high-performance models as crucial tools to support the evaluation process. DT-AdaBoost is an ensemble learning model based on Decision Tree (DT), which is one of the most popular ML algorithms and is widely used to solve classification and regression problems. DT-AdaBoost combines DT using AdaBoost algorithms, which is one of the Boosting ensemble learning methods. Models using AdaBoost can have higher accuracy and are less prone to overfitting due to the fact that during Boosting iterations, the weights of the samples are adjusted based on the performance of the previous round of the learner. For samples that are misclassified, their weights are increased, making the next round of training pay more attention to these misclassified samples.

DT-AdaBoost is generally more accurate and has better generalization ability compared to a single classification model like SVM. Usually, integrated learning models outperform single models in terms of both accuracy and generalization [50]. Research has indicated that integrated learning algorithms yield superior outcomes in dealing with classification imbalance challenges, a notion validated by the results of this study [51]. Compared to RF, DT-AdaBoost has the same DT weak classifier but has a different ensemble learning method. The Bagging used by RF focuses more on the generalization ability of the model, and thus, typically makes the model using this method less accurate than the model using boosting. Furthermore, the Boosting model has the capability to improve accuracy significantly by leveraging an ensemble of multiple classifiers [52]. This allows DT-AdaBoost to achieve better results in practical applications of classification and regression tasks compared to RF or SVM. In this study, DT-Adaboost demonstrated superior performance compared to SVM and RF in both classification and regression scenarios, confirming this result and indicating its tremendous potential for application.

Color evaluation in previous studies often involves sensory assessment [16] or the use of a colorimeter [17], leading to differing levels of subjectivity and time-consuming complexities. The classic machine learning models SVM [53] and RF [54] have been extensively utilized in prior research, demonstrating superior accuracy in certain tasks when compared to alternative models. We aimed to investigate the distinctions in tea quality assessment when utilizing the less frequently employed DT-Adaboost method in comparison to SVM and RF. Additionally, to achieve a swift and precise color evaluation approach, computer vision was utilized to capture images of tea leaves, extract color features, and integrate machine learning models for assessing the color quality of needle-shaped green tea. The DT-Adaboost model exhibited optimal accuracy in both classification and regression tasks, bolstering the model’s stability. Hence, this method offers a novel solution for evaluating the color quality of needle-shaped green tea.

In this study, computer vision technology was employed to capture images of needle-shaped green tea samples, extract 18 color parameters, and when integrated with machine learning algorithms, enabled precise evaluation of the color quality of green tea. This suggests that a targeted feature selection can enhance the accuracy of the model. Previous studies accurately evaluated the shape quality by using computer vision to identify seven varieties of Congou black tea through extraction of morphological parameters [20], which also demonstrates this point. According to the Chinese National Official Standard GB/T 23776-2018 [31], the appearance quality of tea is determined by its raw materials, shape, color, texture, and other characteristics. This indicates that the use of color and shape features to evaluate the appearance quality is not comprehensive. There is limited research on evaluating the quality of raw materials, texture, and other appearance-related characteristics, which indicates a potential direction for future research. Integrating research findings on appearance, color, texture and other pertinent characteristics would represent a novel approach to accurately and objectively assess the visual quality of tea leaves. This can be achieved by leveraging sensor technology in conjunction with algorithm models, thereby reducing reliance on subjective sensory evaluation methods. Additionally, all samples in the dataset consist of needle-shaped green tea sourced from the Chongqing region, representing a limitation of this study. While the overarching evaluation standards for green tea are largely consistent, variances in quality and assessment criteria arise when considering green tea produced across diverse regions and processed using different methods. These discrepancies necessitate that our model functions merely as a benchmark when appraising additional green tea samples. As such, creating an expanded dataset encompassing various regions and processing techniques would facilitate the development of universally applicable evaluation standards for the wider tea market, by providing a more objective and reliable assessment of tea quality. This could aid tea producers and sellers in making informed decisions regarding pricing and marketing strategies. Ultimately, these results could result in heightened consumer satisfaction and loyalty, alongside improved economic performance for the entire tea industry.

## 4. Conclusions

This study utilized computer vision technology and machine learning to develop a model to assess the color of green tea. By creating a dataset comprising 885 images derived from 157 samples of tea, dividing the image into 800 × 800-pixel regions surrounding the central pixel, and then extracting 18 color features, the color data were split into calibration and prediction sets in a 7:3 ratio. Machine learning models such as SVM, RF, and DT-Adaboost were applied to correlate color information with sensory evaluation and construct color-based evaluation models, achieving accurate evaluation of tea color quality. When evaluating the tea color grade and score using machine learning techniques such as classification and regression, the DT-Adaboost model exhibited superior performance. Within the prediction set comprising 266 samples, the grading model trained utilizing the DT-Adaboost algorithm demonstrated a CDR of 98.50%, while the score model exhibited an RPD value of 14.827. This study proved that the combination of computer vision and machine learning models facilitates accurate evaluation of tea color quality. Additionally, the Decision Tree model shows promising prospects for practical implementation. Color characteristics, which reflect consumers’ initial perception, significantly influence their preferences in purchasing green tea. Providing a more objective and reliable assessment of tea quality can aid tea producers and sellers in making informed decisions regarding pricing and marketing strategies. Ultimately, these results can result in heightened consumer satisfaction and loyalty, alongside improved economic performance for the entire tea industry. In future studies, gathering green tea samples from various regions and using diverse processing methods to enhance the dataset, as well as developing assessment models for attributes beyond color, would facilitate the establishment of a more precise and broadly applicable standardized system for assessing green tea quality.

## Figures and Tables

**Table 1:** The classification results obtained using different machine learning models.

Models	Parameters	Accuracy (%)	Precision (%)	F1-Score (%)	CDR (%)
SVM	c = 20, γ = 2	97.74	97.83	97.75	97.74
RF	N = 40, Depth = 10	98.12	98.16	98.12	98.12
DT-Adaboost	N = 70, Depth = 6	98.50	98.56	98.50	98.50

**Table 2:** Performance of different models for sensory score using color data.

Models	Parameters	Calibration Set		Prediction Set		RPD
		Rc	RMSEC	Rp	RMSEP	
SVM	C = 7, γ = 0.3	0.991	0.252	0.983	0.313	7.668
RF	N = 90, depth = 12	0.997	0.136	0.985	0.296	8.122
DT-Adaboost	N = 80, depth = 12	0.999	0.020	0.995	0.162	14.827

## Data Availability

The original contributions presented in the study are included in the article/Supplementary Materials. Further inquiries can be directed to the corresponding author.

## Supplementary Materials

The following supporting information can be downloaded at: https://www.mdpi.com/article/10.3390/foods13162516/s1, Figure S1: Performance assessment of panelists of color evaluation group; Figure S2: The graphical user interface (GUI) software interface and its associated functions; Table S1: Moisture content of needle-shaped green tea samples in Chongqing area.; Table S2: La*b* values for each color score; Table S3: Camera characteristics and lighting conditions.
