# Supplementary material for: Rapid Color Quality Evaluation of Needle-Shaped Green Tea Using Computer Vision System and Machine Learning Models
Source: Foods. 2024 Aug 12;13(16):2516. doi: 10.3390/foods13162516 (PMC11353727; doi:10.3390/foods13162516)
Supplement: Supplementary file 1 [file foods-13-02516-s001.zip › foods-3122075-supplementary.pdf]

Table S1.

Moisture content of needle-shaped green tea samples in Chongqing area.

Origin	Sample moisture content/%							
	1	2	3	4	5	6	7	8
	4.5722	4.0638	5.7188	4.1516	4.7313	4.8201	3.2616	4.1521
	9	10	11	12	13	14	15	16
	3.3976	4.2340	4.9761	4.9596	5.7832	5.1613	3.4235	4.5022
	17	18	19	20	21	22	23	24
	5.3716	5.5381	3.0875	3.0362	4.4291	5.1089	3.9006	5.7901
	25	26	27	28	29	30	31	32
	5.2492	4.7125	5.6384	4.1054	6.2677	3.2720	3.3344	3.2552
	33	34	35	36	37	38	39	40
	4.8852	4.0779	3.7635	3.3456	3.6020	4.1583	4.2512	4.5072
Xiushan County	41	42	43	44	45	46	47	48
	3.1172	3.1384	5.2614	5.6882	5.4396	5.5599	5.0062	5.6510
	49	50	51	52	53	54	55	56
	5.8116	4.7031	6.3520	6.0449	5.8002	4.6775	4.9372	5.9006
	57	58	59	60	61	62	63	64
	4.3082	5.3540	4.7307	6.4874	4.3606	4.7407	4.8694	4.7852
	65	66	67	68	69	70	71	72
	5.6842	4.3135	4.8647	4.8392	5.0980	3.9205	3.9615	4.5556
	73	74	75	76	77	78	79	80
	5.0667	5.3076	5.3820	5.1397	5.6205	4.4357	3.6137	4.6416
	81	82	83	84	85	86		
	5.2118	5.3505	5.0361	5.5525	5.0487	4.9178		
	87	88	89	90	91	92	93	94
	6.4383	5.4566	6.1516	5.9291	5.2814	6.1571	5.7265	5.6406
	95	96	97	98	99	100	101	102
Yongchuan District	5.4447	6.4777	5.4928	5.2236	5.9421	5.1614	5.1882	4.4338
	103	104	105	106	107	108	109	110
	4.7770	5.0981	4.9181	5.4264	4.6425	4.5086	4.2528	2.7539
	111	112						
	4.1820	4.9421						
	113	114	115	116	117	118	119	120
	5.5076	4.8598	5.0253	4.9381	5.8379	5.4850	5.7513	5.4904
	121	122	123	124	125	126	127	128
Wanzhou District	5.2076	5.1821	6.5941	6.6942	4.7545	5.5746	5.6913	6.0389
	129	130	131	132	133	134	135	136
	5.3642	5.5276	5.6057	5.2694	6.9581	5.2408	5.6202	6.1999
	137	138	139	140	141	142	143	144
	6.6775	5.9773	6.0888	3.3416	6.5750	6.5599	5.6878	5.7181

	145	146						
	6.5407	5.7633						
Nanchuan District	147	148	149	150	151	152	153	154
	4.4832	3.9984	3.7404	4.8047	4.5029	4.0823	3.8275	4.1113
	155							
	5.6622							
Wulong District	156							
	6.3742							
Yunyang County	157							
	6.0120							

Table S2.

La^*b^* values for each color score.

Color Score	L	a^*	b^*
15	100	-40	50
14	90	-40	50
13	80	-40	50
12	70	-40	50
11	60	-30	50
10	50	-40	50
9	40	-40	40
8	30	-40	20
7	20	-30	0
6	20	-20	20
5	60	-20	50
4	60	-20	20
3	40	0	50
2	10	-40	20
1	10	-20	10

Table S3.

Camera characteristics and lighting conditions.

**Characteristic**	**Parameter**
camera altitude	300 mm
Light altitude	260 mm
luminosity intensity	100 Lux
Image size	2590×1944
Aperture	F/4.0
Exposure time	1/30 s
White balance	R1.40/G1.00/B1.30
Image type	Bmp

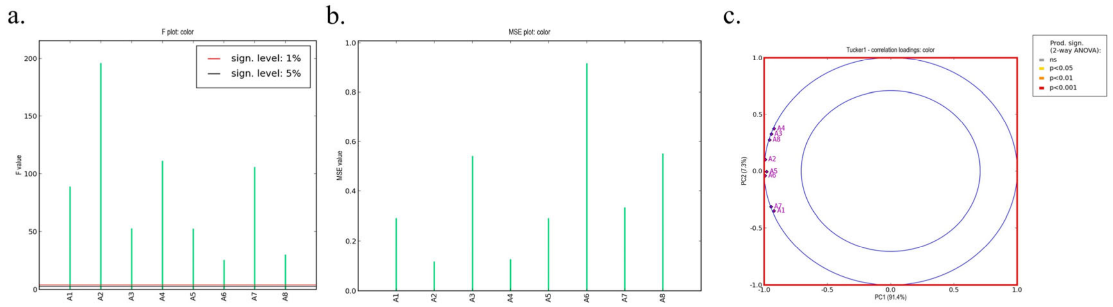

Figure S1.

Performance assessment of panelists of color evaluation group:(a) F value, Reflect the panelist's ability to distinguish samples;(b) MSE value, Reflect the repeatability of panelist evaluation results;(c) Tucker-1 value, Reflecting the consistency of the evaluation group;

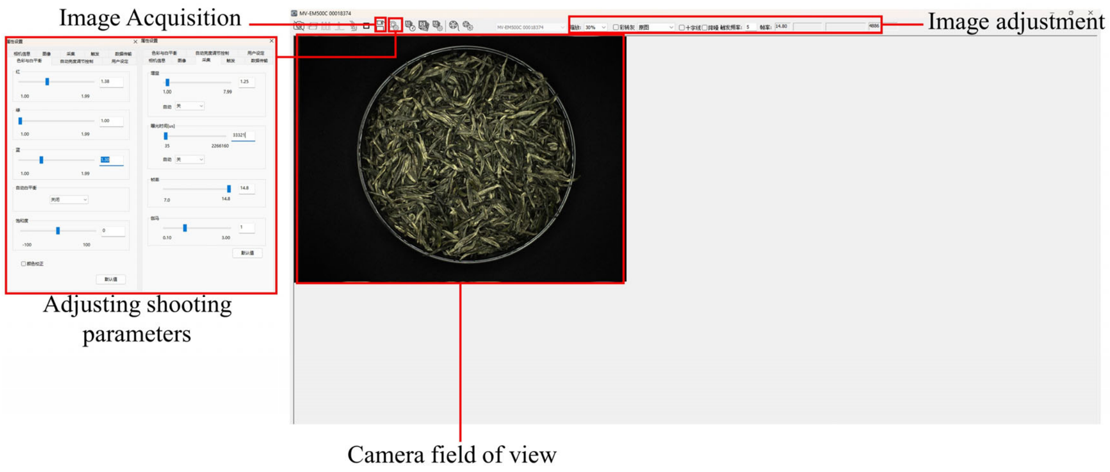

Figure S2.

The graphical user interface (GUI) software interface and its associated functions.
